# Vasopressors for the Treatment of Septic Shock: Systematic Review and Meta-Analysis

**DOI:** 10.1371/journal.pone.0129305

**Published:** 2015-08-03

**Authors:** Tomer Avni, Adi Lador, Shaul Lev, Leonard Leibovici, Mical Paul, Alon Grossman

**Affiliations:** 1 Medicine E, Rabin Medical Center, Beilinson Hospital, Petah-Tikva, Israel and Sackler Faculty of Medicine, Tel-Aviv University, Tel-Aviv, Israel; 2 Intensive Care Unit, Rabin Medical Center, Beilinson Hospital, Petah-Tikva, Israel and Sackler Faculty of Medicine, Tel-Aviv University, Tel-Aviv, Israel; 3 Infectious diseases Unit, Rambam Medical Center and Rappaport Faculty of Medicine, Tehnion—Israel Institute of Technology, Haifa, Israel; San Raffaele Scientific Institute, ITALY

## Abstract

**Objective:**

International guidelines recommend dopamine or norepinephrine as first-line vasopressor agents in septic shock. Phenylephrine, epinephrine, vasopressin and terlipressin are considered second-line agents. Our objective was to assess the evidence for the efficiency and safety of all vasopressors in septic shock.

**Methods:**

Systematic review and meta-analysis. We searched electronic database of MEDLINE, CENTRAL, LILACS and conference proceedings up to June 2014. We included randomized controlled trials comparing different vasopressors for the treatment of adult patients with septic shock. Primary outcome was all-cause mortality. Other clinical and hemodynamic measurements were extracted as secondary outcomes. Risk ratios (RR) and mean differences with 95% confidence intervals (CI) were pooled.

**Results:**

Thirty-two trials (3,544 patients) were included. Compared to dopamine (866 patients, 450 events), norepinephrine (832 patients, 376 events) was associated with decreased all-cause mortality, RR 0.89 (95% CI 0.81-0.98), corresponding to an absolute risk reduction of 11% and number needed to treat of 9. Norepinephrine was associated with lower risk for major adverse events and cardiac arrhythmias compared to dopamine. No other mortality benefit was demonstrated for the comparisons of norepinephrine to epinephrine, phenylephrine and vasopressin / terlipressin. Hemodynamic data were similar between the different vasopressors, with some advantage for norepinephrine in central venous pressure, urinary output and blood lactate levels.

**Conclusions:**

Evidence suggests a survival benefit, better hemodynamic profile and reduced adverse events rate for norepinephrine over dopamine. Norepinephrine should be regarded as the first line vasopressor in the treatment of septic shock.

## Introduction

Septic shock and severe sepsis are a grave consequence of infection. Septic shock accounts for about 9% of admissions, and is the most common cause of death in intensive care units (ICUs) [1,2]. The mortality rate reported is 40 to 60% [1,3]. Septic shock is defined by the ACCP/SCCM as the need for vasopressors to reverse sepsis-induced hypotension [4]. The concept of "early goal directed therapy" was developed in order to set early hemodynamic goals. The goals include central venous or mixed venous saturation (ScvO_2_) higher than 70%, mean arterial pressure (MAP) ≥ 65 mmHg, central venous pressure (CVP) > 8–12 mmHg, and urine output > 0.5 ml/kg/hr within 6 hours of initiation of therapy [5]. Later the concept of lactate clearance was added to the goals as an alternative or addition to the ScvO_2_ [6]. Thus, hemodynamic outcomes are regarded as surrogate markers for survival, the ultimate goal of treatment.

No study to date has demonstrated a statistically significant survival benefit of one vasopressor over another. Therefore, the choice of vasopressor in septic shock is rather empiric. The Surviving Sepsis Campaign recommends norepinephrine or dopamine as the first-choice vasopressor agent followed by epinephrine in patients who respond poorly to dopamine or norepinephrine [7,8].

In this systematic review we aimed to examine the evidence on the relative clinical, hemodynamics and safety of any vasopressors in the treatment of septic shock, in order to guide future trials and treatment guidelines.

## Methods

### Inclusion criteria

Randomized controlled trials (RCTs) and randomized crossover trials regardless of publication status were included. We included trials assessing adult patients with septic shock and sepsis with multiple organ dysfunction syndrome diagnosed using established criteria [4], or an accepted definition of severe sepsis and end organ damage caused by hypoperfusion. In studies that recruited patients with shock due to many causes, we extracted data for septic patients where possible. The intervention assessed was vasopressor versus a different vasopressor, a combination of vasopressors, placebo or no vasopressor. The following vasopressors were included: dopamine, norepinephrine, epinephrine, phenylephrine, vasopressin and terlipressin.

We regarded vasopressin and terlipressin as non-adrenergic vasopressors. Trials that added open-label vasopressor(s) were included if it was applied to both arms of intervention. Studies comparing interventions limited to inotrope agents were excluded. We excluded studies that assessed different dosages or schedules of the same vasopressors.

### Outcomes assessed

The primary outcome was all-cause mortality at 28 days following randomization, or when lacking these data, all-cause mortality as reported by the authors. Secondary outcomes included length of ICU stay and / or hospital stay in patients discharged alive; ventilator free days; vasopressor free days; hemodynamic profiles of patients at the first hour (or first measuring point), and at the 6^th^ hour (or second measuring point); and adverse events (AEs) (major AEs, arrhythmias, myocardial infarction, stroke, internal organ ischemic damage, and local extravasation or skin necrosis). The hemodynamic profile consisted of CVP, MAP, ScvO_2_, urinary output, blood lactate levels, cardiac index (CIX), systemic vascular resistance index (SVRI), heart rate, and oxygen delivery index (VIO_2_); we also collected measurements of splanchnic blood flow, oxygen delivery, oxygen consumption and visceral CO_2_ difference as measured by catheter sampling or tonometry. In studies that allowed crossover between arms, we included data regarding the first randomization only, if available. Mortality was not extracted from crossover studies. Outcome measures were collected on an intention-to-treat basis. Where such data was not presented, per-protocol results were used. We also compared results for adrenergic vasopressors versus non-adrenergic vasopressors.

### Search methods

We searched MEDLINE, CENTRAL, LILACS and conference proceedings (International Symposium on Intensive Care and Emergency Medicine), up to June 2014. We also hand-searched all references of included studies, and previous meta-analyses for more trials. The words "vasopressor" and vasopressor names and their MESH terms were crossed with the terms "hypotension", "circulatory failure", "shock", "sepsis" or "bacteremia" and with the Cochrane highly sensitive filter for RCTs [9]. No language restrictions were used. Authors were contacted to complement data by e-mail and phone calls.

### Data collection

Two reviewers independently inspected each reference identified by the search, scanned full-texts of relevant studies, applied the inclusion criteria and extracted the data. Disagreements in data extraction were resolved by discussion with a third reviewer. Risk of bias was assessed in duplicate using domain-based evaluation, classifying studies primarily according to the risk of non-random allocation of patients to the intervention arm, i.e. allocation concealment and sequence generation. These were graded as adequate, unclear and inadequate as recommended in The Cochrane Handbook [9]. Additional domains assessed included blinding, incomplete outcome data reporting, ethics committee, patient consent and industrial sponsorship.

### Data analysis

For binary data, individual study results are expressed as RR with 95% CI. For continuous outcomes we extracted end-value means with standard deviations (SD), as data for change from baseline was unavailable. In studies that reported median with interquartile ranges, we converted the reported values to means assuming a normal distribution [10]. RRs and mean differences were pooled using a fixed effect model (Mantel-Haenszel method) (Review Manager [RevMan], version 5.3 for Windows, The Cochrane Collaboration, Oxford, UK). Heterogeneity was defined by a chi-square test of heterogeneity <0.1 or an I^2^ measure of inconsistency >40%. If significant heterogeneity was identified, we used random effect model (REM). We explored potential sources of heterogeneity: trials published before versus after the year 2004 (surviving sepsis campaign), trials conducted in developed versus developing countries, and the adequacy of allocation concealment and blinding

## Results

**Fig 1:**
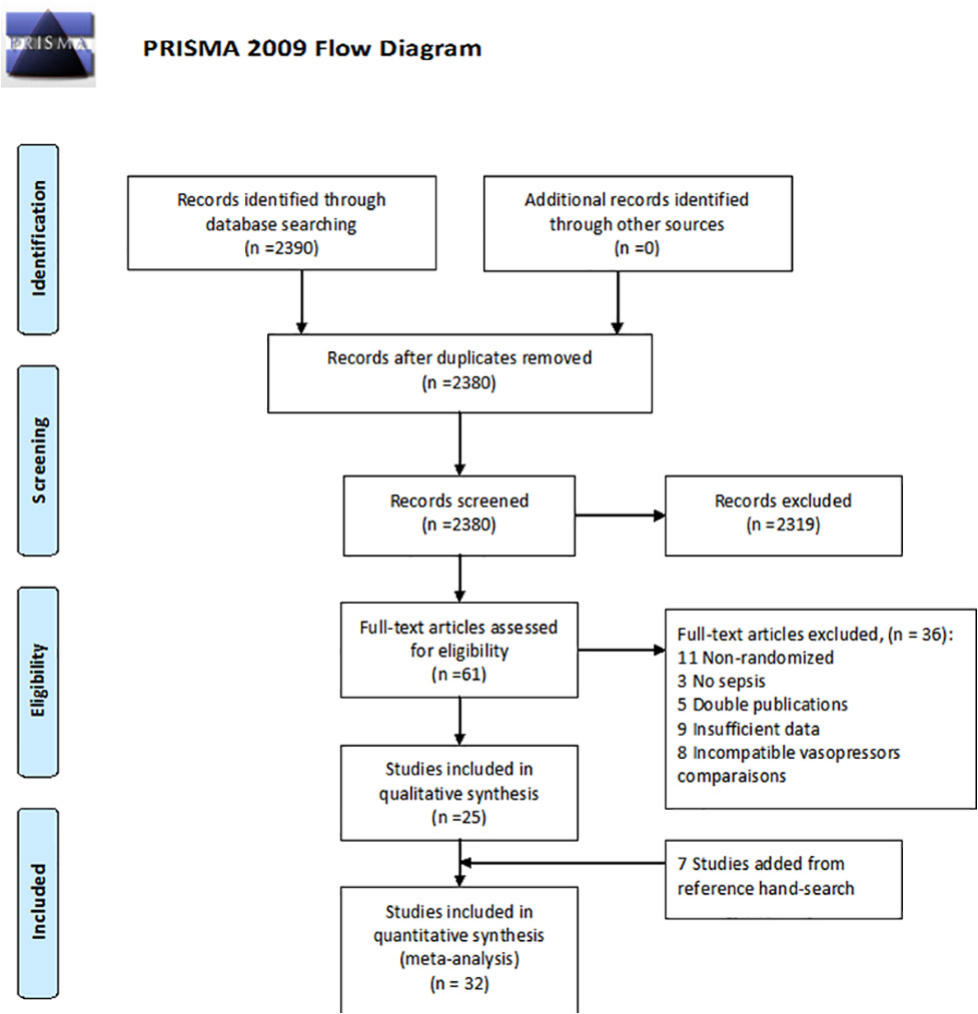
Study flow chart.

**Fig 2:**
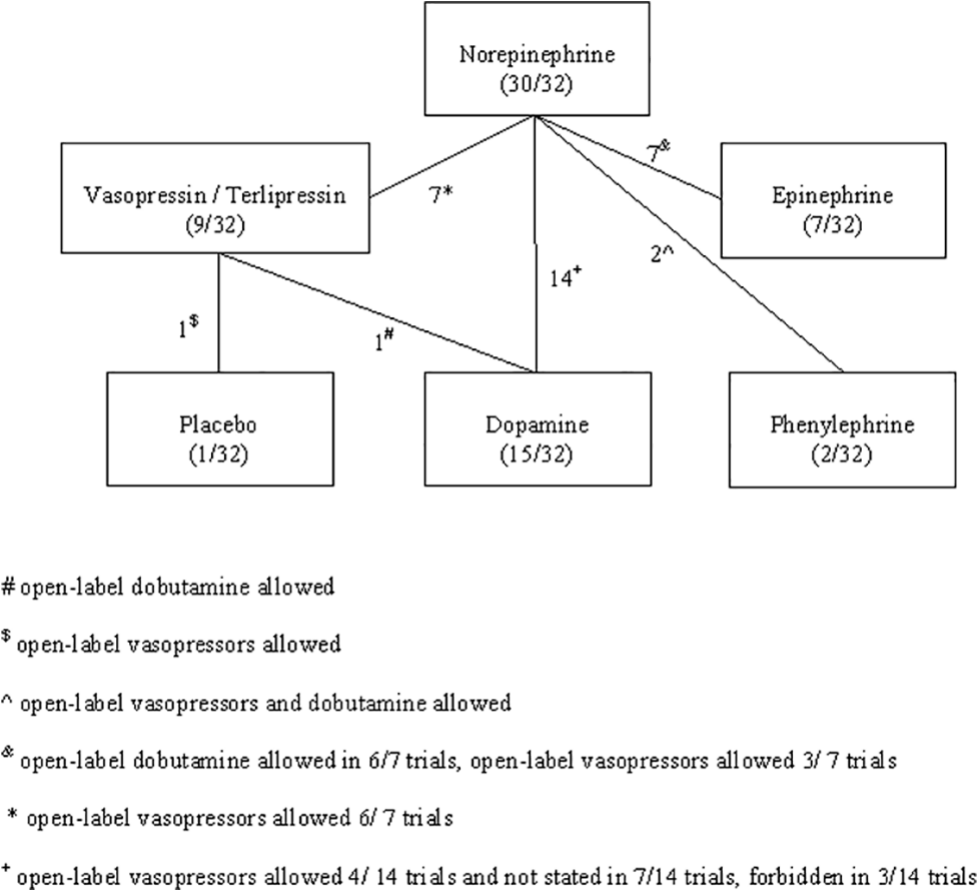
Vasopressors arms comparisons.

The search yielded 2,380 publications of which 61 were potentially relevant. Thirty-six studies were excluded (Fig 1), and 7 studies were added from reference hand searching. Altogether, 32 studies [11–42] were included (all published in peer-review journals). The trials were published in the years 1989–2012 and recruited 3,544 patients (median 50 patients, Table 1 for trials’ characteristics). Five trials were crossover [16–18,37,41] and the remaining were parallel RCTs. The comparisons assessed in included trials are summarized in Fig 2. The main comparisons were between norepinephrine and dopamine (14 trials, including 2 crossover trials) and norepinephrine and epinephrine (7 trials including 3 crossover trials). One trial compared vasopressin to placebo [24]; Additional open-label vasopressors could be added to both study arms equally in 16/32 trials (Fig 2). Eight trials were designed to measure clinical endpoints as the primary outcome, while 26 trials, including all crossover trials, measured hemodynamic data (crossover time at 45–220 minutes). The follow up duration was not stated in 21/32 trials; When stated, follow-up ranged from length of the ICU stay (3 trials) to 90 days (median 28 days). The common sources of infection were respiratory (34.6% of included patients) and intra-abdominal (25.1%). Steroid administration was reported in 5 studies, and given to 31–100% of all patients (median 74%), (Table 1). The amount of fluids that was given for the resuscitation was reported in 7/32 trials and varied considerably (median 150 ml/hr, range 45–300 ml/hr). Another 10 trials reported a protocol based fluid resuscitation and titration by CIX. The weighted mean all-cause mortality was 45.0% (SD 16.2%, range 16.7–88.7%) and was not significantly correlated with publication year (Spearman’s rho -0.260, p = 0.24, 32 studies, Table 1).

**Table 1:** Trials characteristics.

Study ID	Country, SC / MC^1^	Intervention	Primary outcome	N patients	Mean age	Mean MAP at baseline (mmHg)	Mean lactate at baseline (mmol/l)	Mean cardiac index (l/min/m^2^)	APACHE II / SAPS-2 / SOFA score at baseline ^2^	28 days, All-cause mortality	Mortality in longest follow up, follow-up duration
Agrawal 2011 [11]			hemodynamic								NS
	India, SC	Norepinephrine		25	52	NS	NS	5.45	25 (A)	NS	NS
		Dopamine		25	54	NS	NS	5.28	24 (A)	NS	NS
Albanese 2005 [12]			hemodynamic								NS
	France, SC	Norepinephrine		10	65	54	NS	5.1	29 (A)	40%	NS
		Terlipressin		10	66	54	NS	5.0	28 (A)	50%	NS
Annane 2007 [13]			survival								90 days
	France, MC	Norepinephrine + Dobutamine		169	60	68	3.3	NS	52 (S)	34%	50%
		Epinephrine		161	65	70	2.9	NS	54 (S)	39%	52%
Chen 2012 [14]			hemodynamic								NS
	China, SC	Norepinephrine		40	NS	NS	NS	NS	NS	22%	NS
		Dopamine		40	NS	NS	NS	NS	NS	27%	NS
De Backer 2003 [16]			hemodynamic							80%	NS
	Belgium, SC	Norepinephrine crossover		5	NS	NS	NS	NS	19 (A)		
		Epinephrine crossover		5	NS	NS	NS	NS	19 (A)		
De Backer 2010 [15]			survival								12 months
	Belgium, Austria, Spain, MC	Norepinephrine		502	NS	NS	NS	NS	NS	49%	NS
		Dopamine		542	NS	NS	NS	NS	NS	53%	NS
Duranteau 1999 [17]			hemodynamic							58%	NS, NS
	France, SC	Norepinephrine crossover		4	54	NS	NS	NS	NS		
		Norepinephrine + Dobutamine crossover		4	54	NS	NS	NS	NS		
		Epinephrine crossover		4	54	NS	NS	NS	NS		
Guérin 2005 [18]			hemodynamic							16%	NS, NS
	France, SC	Norepinephrine crossover		6	40	56.8	2.3	4.07	14 (A)		
		Dopamine crossover		6	40	56.8	2.3	4.07	14 (A)		
High 2008[19]			hemodynamic		56						NS
	China, SC	Norepinephrine		23		NS	NS	NS	NS	39%	NS
		Dopamine		21		NS	NS	NS	NS	38%	NS
Jain 2010 [20]			hemodynamic								NS
	India, SC	Norepinephrine		27	42	68	NS	5.0	17.6 (A)	55%	NS
		Phenylephrine		27	45	69	NS	4.8	19.1 (A)	59%	NS
Lauzier 2006 [21]			hemodynamic								NS
	France, Canada, MC	Norepinephrine		10	58.1	68	3.33	4.4	23.5 (A)	33%	NS
		Vasopressin		13	51.2	72	2.87	4.6	22.8 (A)	23%	NS
Levy 1997[22]			hemodynamic								NS
	France, SC	Norepinephrine + Dobutamine		15	56	60	3.1	4.0	25.6 (A)	53%	NS
		Epinephrine		15	54	60	3.1	4.0	24.5 (A)	60%	NS
Liu 2010 [23]			hemodynamic		43						NS
	China, SC	Norepinephrine		25		NS	4.9	NS	16 (A)	32%	NS
		Dopamine		25		NS	4.6	NS	17 (A)	48%	NS
Malay 1999 [24]			hemodynamic								NS
	US, SC	Placebo		5	56	64	NS	5.6	26 (A)	40%	NS
		Vasopressin		5	53	66	NS	4.3	27 (A)	0	NS
Marik 1994 [25]			hemodynamic								NS
	US, SC	Norepinephrine		10	46	65	1.8	4.2	18 (A)	50%	NS
		Dopamine		10	46	63	2.2	4.2	17 (A)	60%	NS
Marthur 2007 [26]			hemodynamic								NS
	India, SC	Norepinephrine		25	52	NS	NS	5.4	25.6 (A)	56%	NS
		Dopamine		25	54	NS	NS	5.2	24.5 (A)	76%	NS
Martin 1993 [27]			hemodynamic								NS
	France, SC	Norepinephrine		16	52	54	4.8	5.4	31 (A)	43%	NS
		Dopamine		16	53	53	4.8	5.3	30 (A)	62%	NS
Morelli 2008 [30]			hemodynamic								NS
	Italy, SC	Norepinephrine		16	70	55	2.8	4.4	55 (S)	56%	NS
		Phenylephrine		16	70	54	2.8	4.3	57 (S)	62%	NS
Morelli 2009 [29]			hemodynamic								NS
	Italy, SC	Norepinephrine		15	64	54	3.1	4.0	58 (S)	66%	NS
		Vasopressin		15	66	53	3.0	4.0	60 (S)	53%	NS
		Terlipressin		15	67	53	3.1	4.0	62 (S)	46%	
Morelli 2011[28]			hemodynamic								NS
	Italy, SC	Norepinephrine		20	66	71	2.5	4.0	54 (S)	NS	NS
		Vasopressin		20	61	72	2.3	4.0	53 (S)	NS	NS
		Terlipressin		20	65	71	1.8	3.8	50 (S)	NS	NS
Myburgh 2008 [31]			hemodynamic								90 days
	Australia, MC	Norepinephrine		82	60	66	2.4	NS	22.7 (A)	29%	36.5%
		Epinephrine		76	58	65	2.7	NS	23.4 (A)	22%	31%
Patel 2002 [32]			hemodynamic								NS
	Canada, SC	Norepinephrine		11	68	68	NS	5.0	24 (A)	NS	NS
		Vasopressin		13	68	69	NS	4.8	22 (A)	NS	NS
Patel 2010 [33]			survival								28 days
	US, MC	Norepinephrine		118	NS	NS	NS	NS	27 (A)	43%	43%
		Dopamine		134	NS	NS	NS	NS	28 (A)	50%	50%
Plotkin 2007 [34]			survival								NS
	Russia, SC	Dopamine		33	40	NS	3.2	2.8	24 (A)	90%	NS
		Terlipressin		41	40	NS	3.6	2.8	24 (A)	73%	NS
Ruokonen 1993 [35]			hemodynamic								NS
	Finland, SC	Norepinephrine		5	44	55	2.1	4.4	13.3 (A)	80%	NS
		Dopamine		5	46	59	1.3	4.1	13.3 (A)	60%	NS
Russell 2008 [36]			survival								90 days
	US, Australia, Canada, MC	Norepinephrine		395	61	73	3.5	NS	27.1 (A)	39%	49%
		Vasopressin		404	59	72	3.5	NS	27 (A)	35%	44%
Schreuder 1989 [37]			hemodynamic							80%	NS, NS
	The Netherlands, SC	Norepinephrine crossover		5	57	56	2.3	4.07	NS		
		Dopamine crossover		5	57	56	2.3	4.07	NS		
Seguin 2002 [38]			hemodynamic								NS
	France, MC	Norepinephrine + Dobutamine		11	70	51	5.3	3.0	62 (S)	45%	NS
		Epinephrine		11	65	54	3.9	3.2	57 (S)	36%	NS
Svoboda 2012 [39]			hemodynamic								90 days
	Czech Republic, SC	Norepinephrine		13	75	71	NS	NS	18 (SO)	76%	92%
		Terlipressin		17	70	74	NS	NS	18 (SO)	94%	94%
Wu 2010 [40]			hemodynamic		55						NS
	China, SC	Norepinephrine		23		52	5.9	4.4	22 (A)	30%	NS
		Dopamine		23		50	6.1	4.3	21 (A)	39%	NS
Zhou 2002 [41]			hemodynamic							58%	NS
	China, SC	Norepinephrine crossover		4	NS	60	2.6	4.9	22 (A)		NS
		Norepinephrine + Dobutamine crossover		4	NS	60	2.6	4.9	22 (A)		NS
		Epinephrine crossover		4	NS	60	2.6	4.9	22 (A)		NS
Zhuangyu 2011 [42]			survival								NS
	China, SC	Norepinephrine		45	54	71	NS	NS	24 (A)	28%	NS
		Dopamine		45	55	74	NS	NS	25 (A)	31%	NS

All results present in mean (or median if not reported).

^1^ MC–multicenter, SC–single-center.

^2^ (A)—APACHE II, (S)–SAPS-2, (SO)–SOFA score.

### Risk of bias assessment

Risk of bias assessment is detailed in Table 2. Low risk sequence generation and allocation concealment were reported in 16/32 (50%) and 13/32 (40.6%) trials, respectively. One study was triple blinded [15], 8 trials were double blinded [13, 24, 26, 27, 30–32, 36], 2 were single blinded [20, 34] and the others were open-labeled or not stated. Results were analysed by intention-to-treat in all trials. Informed consent and ethical committee approval were described in 26/32 and 25/32 trials, respectively. Industrial sponsorship was stated in one trial [39]. Definitions of sepsis and septic shock for inclusion criteria, were described as suggested by the ACCP/SCCM consensus or similar by 30/32 (93.7%) of all studies.

**Table 2:** Risk of bias assessment.

Study ID	Sequence generation	Allocation concealment	Blinding	Ethical committee / informed consent	ITT analysis of primary outcome	Incomplete data reporting	Valid definition of septic shock
Agrawal 2011 [11]	Unclear	Unclear	Open	Yes / Yes	Yes	Unclear	Yes
Albanese 2005 [12]	Low	Unclear	Open	Yes / Yes	Yes	Unclear	Yes
Annane 2007 [13]	Low	Low	Double	Yes / Yes	Yes	No	Yes
Chen 2012 [14]	Unclear	Unclear	Open	Unclear / Unclear	Yes	Unclear	Yes
De Backer 2003 [16]	Unclear	Unclear	Open	Yes / Yes	Yes	Unclear	Yes
De Backer 2010 [15]	Low	Low	Triple	Yes / Yes	Yes	No	Yes
Duranteau 1999 [17]	Unclear	Unclear	Open	Yes / Yes	Yes	Unclear	Yes
Guérin 2005 [18]	Unclear	Unclear	Open	Yes / Yes	Yes	Unclear	Yes
High 2008[19]	Unclear	Unclear	Open	Unclear / Unclear	Yes	Unclear	No
Jain 2010 [20]	Low	Low	Single	Yes / Yes	Yes	Unclear	Yes
Lauzier 2006 [21]	Low	Low	Open	Yes / Yes	Yes	Unclear	Yes
Levy 1997[22]	Low	Low	Open	Yes / Yes	Yes	Unclear	Yes
Liu 2010 [23]	Unclear	Unclear	Open	Unclear / Unclear	Yes	Unclear	Yes
Malay 1999 [24]	Low	Unclear	Double	Yes / Yes	Yes	Unclear	Yes
Marik 1994 [25]	Low	Low	Open	Yes / Unclear	Yes	Unclear	Yes
Marthur 2007 [26]	Unclear	Unclear	Double	Yes / Yes	Yes	Unclear	Yes
Martin 1993 [27]	Low	Low	Double	Yes / Yes	Yes	Unclear	Yes
Morelli 2008 [30]	Low	Low	Double	Yes / Yes	Yes	No	Yes
Morelli 2009 [29]	Low	Low	Open	Yes / Yes	Yes	No	Yes
Morelli 2011[28]	Unclear	Unclear	Open	Yes / Yes	Yes	Unclear	Yes
Myburgh 2008 [31]	Low	Low	Double	Yes / Yes	Yes	No	Yes
Patel 2002 [32]	Low	Unclear	Double	Yes / Yes	Yes	Unclear	Yes
Patel 2010 [33]	High	Unclear	Open	Yes / Yes	Yes	No	Yes
Plotkin 2007 [34]	Unclear	Unclear	Single	Unclear / Unclear	Yes	Unclear	Yes
Ruokonen 1993 [35]	Unclear	Unclear	Open	Yes / Yes	Yes	Unclear	Yes
Russell 2008 [36]	Low	Low	Double	Yes / Yes	Yes	No	Yes
Schreuder 1989 [37]	Unclear	Unclear	Open	Yes / Yes	Yes	Unclear	Yes
Seguin 2002 [38]	Low	Low	Open	Yes / Yes	Yes	Unclear	Yes
Svoboda 2012 [39]	Low	Low	Open	Yes / Yes	Yes	Unclear	Yes
Wu 2010 [40]	Unclear	Unclear	Open	Unclear / Unclear	Yes	Unclear	Yes
Zhou 2002 [41]	Unclear	Unclear	Open	Yes / Yes	Yes	Unclear	Yes
Zhuangyu 2011 [42]	Unclear	Unclear	Open	Unclear / Unclear	Yes	Unclear	No

### Primary outcome

**Fig 3:**
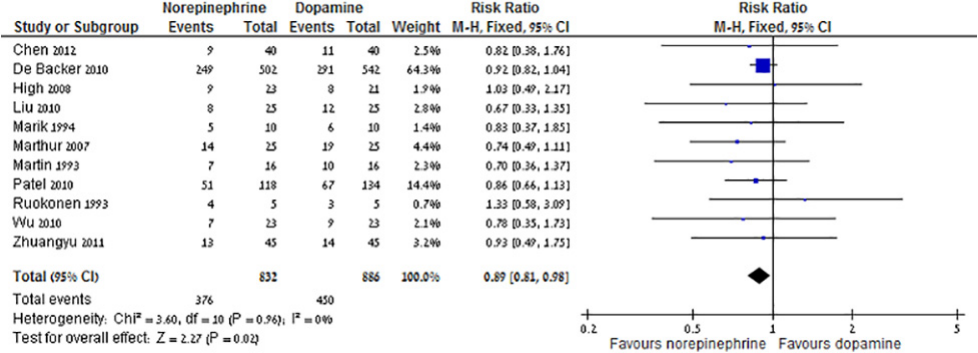
Norepinephrine versus dopamine, 28 days all-cause mortality.

Mortality was reported in eleven trials that compared norepinephrine to dopamine. Funnel plot did not detect high risk of publication bias (S1 Fig). Norepinephrine was associated with reduced all-cause mortality (RR 0.89, 95% CI 0.81–0.98, I^2^ = 0%), corresponding to an absolute risk reduction of 11% and number needed to treat of 9 (Fig 3). Excluding De Backer's study from 2010 [15] which contributed 64% of the weight, did not alter the results, RR 0.84 (95% CI 0.70–0.99, I^2^ = 0%, n = 10), as the exclusion of other trials. Sensitivity analysis restricted to trials from the developed world, trials published after 2004, trials with adequate allocation concealment and generation and blinding did not alter this result. Analysis based on trials whose primary outcome was clinical [15, 33, 42] revealed the same trend for reduced mortality with norepinephrine that was not statistically significant (RR 0.91 95% CI 0.82–1.02).

There was no statistically significant mortality benefit with norepinephrine over epinephrine (RR 0.96, 95% CI 0.77–1.21, I^2^ = 0%, n = 4). Excluding one trial that did not add dobutamine to norepinephrine did not alter the results (RR 0.89, 95% CI 0.69–1.14, I^2^ = 0%, n = 3). There was no statistically significant mortality benefit with norepinephrine over vasopressin / terlipressin RR 1.07 (95% CI 0.91–1.26, I^2^ = 0%, n = 5), and over phenylephrine RR 0.92 (95% CI 0.64–1.32, I^2^ = 0%, n = 2). Results for the comparison of dopamine to terlipressin, and vasopressin to placebo were not pooled (one trial for each comparison, no change in mortality ratio within each trial). When compared to all other treatments, norepinephrine and vasopressin were not associated with a benefit in mortality (RR 0.96, 95% CI 0.86–1.04, I^2^ = 0%, n = 16, and RR 1.01, 95% CI 0.88–1.15, I^2^ = 14%, n = 8, respectively). Comparing adrenergic to non-adrenergic vasopressors resulted in no benefit in mortality for either groups (RR 1.17, 95% CI 0.90–1.51, I^2^ = 10%, n = 7). The pre-defined sensitivity analyses did not alter these results.

### Secondary outcomes

#### Length of ICU stay, days free of vasopressors / ventilation and AEs

**Fig 4:**
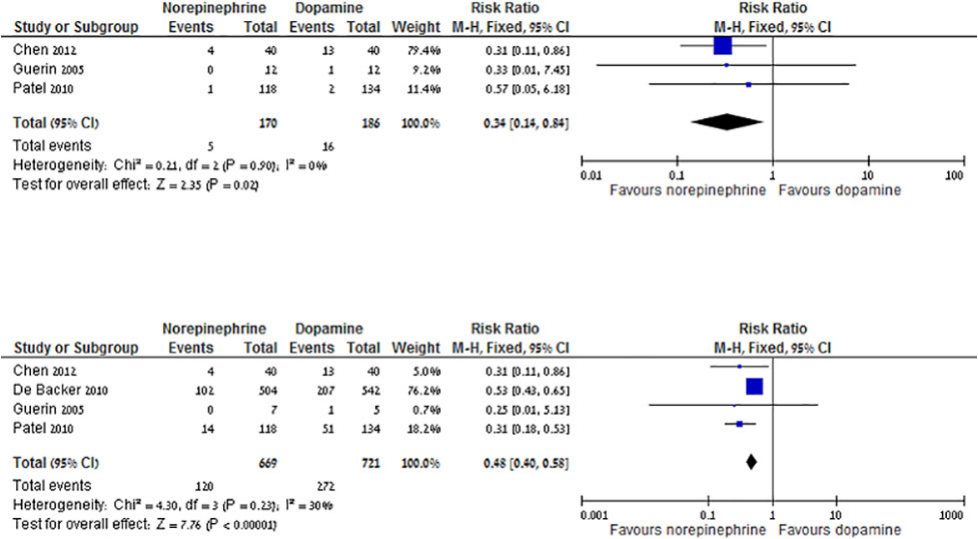
a. Norepinephrine versus dopamine, major adverse events. b. Norepinephrine versus dopamine, cardiac arrhythmias.

ICU stay was reported in 6 trials, all compared norepinephrine to other vasopressors (median 13 days range 7–25). No advantage was found for the use of norepinephrine over other vasopressors (mean difference 1.01 days, 95% CI -0.65–2.66, I^2^ = 0%) (S2 Fig). Hospital stay in patients discharged alive was reported in 3 trials, all compare norepinephrine to other vasopressors (median 15 days, range 7–52). No advantage was found for the use of norepinephrine. Only two trials reported data for days free of ventilation and days free of vasopressor support, therefore we did not pool these results. Eight trials reported major AEs, which included life threatening tachyarrhythmias (37 events, 3 trials), any arrhythmias (475 events, 7 trials), myocardial ischemia or arrest (80 events, 5 trials), stroke (13 events, 2 trials), internal organ or limb ischemia (126 events, 4 trials) and other (9 events, 1 trial). Major AEs were decreased with norepinephrine in comparison to dopamine, RR 0.34 (95% CI 0.14–0.84, I^2^ = 0%, n = 3, Fig 4). Arrhythmias, were significantly decreased with norepinephrine compared to dopamine, RR 0.48 (95% CI 0.40–0.58, I^2^ = 30%, n = 4, Fig 4). Compared with vasopressin / terlipressin, norepinephrine was not associated with a statistically significant change in major AEs and arrhythmias. Due to lack of reported data, we did not pool other AEs separately. Data for AEs with epinephrine and phenylephrine was sparse and did not allow pooled meta-analysis.

#### Hemodynamic data–ScvO2, urinary output, lactate clearance, MAP, CVP

Forest plots of the presented data are available as Supporting Information (S2 Fig). Clinical and hemodynamic measurements at the beginning of the interventions, varied significantly between the trials (S1 Table). No vasopressor had a statistically significant effect of the MAP at any measurement point, compared to other vasopressor. CVP was higher with norepinephrine at the first measuring point compared to all other vasopressors by a mean of 0.84 mmHg (95% CI 0.16–1.51, I^2^ = 0%, n = 7). No vasopressor had a statistically significant effect on ScvO_2_ levels compared to other vasopressors, at any measurement point. Lactate levels in the first measuring point were lower with norepinephrine compared to vasopressin / terlipressin by a mean 0.23 mmol/l (95% CI 0.13–0.34, I^2^ = 0, n = 6). Compared to all other treatment, norepinephrine was not associated with decreased lactate levels at the first measuring point. No other effect on lactate levels was demonstrated with either comparison and by the pre-defined sensitivity analysis. Norepinephrine was associated with an increase in urine output compared to dopamine by a mean of 0.31 ml/kg/min (95% CI 0.12–0.49, I^2^ = 0%, n = 4). There were no other statistically significant results comparing urine output between other vasopressors. **Hemodynamic data—other measurements**

Forest plots of the presented data are available at Supporting Information (S2 Fig). Fourteen trials (43.5%) included only patients with high baseline CIX (defined as CIX > 3.5 l/min/m^2^ by authors). At the first measuring point, CIX decreased with norepinephrine compared to dopamine by a mean of 0.35 l/min/m^2^ (95% CI 0.22–0.48, I^2^ = 18%, n = 7), to epinephrine by a mean of 0.87 l/min/m^2^ (95% CI 0.57–1.16, I^2^ = 0%, n = 4), and to all other vasopressors by a mean 0.7 l/min/m^2^ (95% CI 0.42–0.97, I^2^ = 42%, REM, n = 14). Heart rate was decreased with norepinephrine compared to dopamine in the first measuring point by a mean difference of 18.76 beats per minute (95% CI 9.76–27.76. I^2^ = 96%, REM, n = 8). When comparing norepinephrine to all other interventions, a decreased heart rate at the first measurement point of 8.92 beats per minute was noted (95% CI 1.6–16.23, I^2^ = 97%, REM, n = 19). One-hour SVRI measurements were higher with norepinephrine compared to dopamine and vasopressin / terlipressin by a mean difference of 192.82, 95% CI 60–325, I^2^ = 86%, REM, n = 8 and 196.56 (95% CI 4.4–3.88, I^2^ = 0%, n = 3, respectively).

Oxygen delivery index in the first measuring point were decreased with norepinephrine compared to other vasopressors by a mean of 14.06 ml/min/m^2^ (95% CI 0.16–27.9, I^2^ = 59%, REM, n = 10). First measuring point splanchnic CO_2_ difference was lower with norepinephrine in comparison to epinephrine by a mean difference of 3.74 (95% CI 1.82–5.66, I^2^ = 42%, REM, n = 4). There were insufficient data to compare vasopressors with regard to splanchnic blood flow and splanchnic oxygen delivery index.

## Discussion

The pooled evidence summarized in this meta-analysis shows absolute reduction of 11% in 28-days all-cause mortality with norepinephrine compared with dopamine corresponding to number needed to treat of 9. Dopamine resulted in more than twice the risk for major AEs including a twofold increase in the risk for cardiac arrhythmias. The hemodynamic profile of norepinephrine was also more favorable than the other vasopressors, resulting in decreased lactate levels, increased CVP and urine output in comparison to the other vasopressors. Further benefits of norepinephrine included reduced CIX and heart rate, elevated SVRI and reduced VIO_2_ and splanchnic CO_2_ difference.

We did not demonstrate mortality benefit with norepinephrine over epinephrine, phenylephrine and vasopressin / terlipressin or between the other comparisons, although a trend towards reduced mortality with norepinephrine was seen in all comparisons. Clinical outcomes other than mortality were seldom reported and therefore it was not possible to get strong evidence for length of ICU / hospital stay and ventilator / vasopressor free days. AEs were also reported only by 15/32 trials. The lower rates of cardiac arrhythmia and major AEs with norepinephrine compared to dopamine might account for the reduced mortality rates observed with norepinephrine.

Data regarding clinical and hemodynamic measurement were sparse and inconsistent in methodology and time of report. Although considered as targets for early goal directed therapy, most trials did not report on the variables included in the algorithm (including both studies that were published before and after 2004 with the publication of the surviving sepsis campaign [8]). The concept of early goal directed therapy was challenged recently in a large RCT [43], in which no mortality benefit with protocol based resuscitation was observed compared with usual care. Thus, obtaining the predefined hemodynamic goal with either vasopressor may not be translated to any clinical outcome change.

No statistically significant changes were observed between vasopressin / terlipressin and all adrenergic vasopressors (norepinephrine, dopamine) regarding mortality and AEs. One of the rationales for the use of non-adrenergic vasopressors is relative vasopressin deficiency in patients with septic shock [44] and the hypothesis that vasopressin can reduce the need for catecholamines and AEs associated with adrenergic stimulation. We did not observe a reduction in AEs, but most patients in the vasopressin arm received norepinephrine at randomization and at least for a part of the trial period as open-label vasopressor.

The use of vasopressors and inotropes for several conditions were addressed previously in systematic reviews and meta-analysis. A Cochrane systematic review [45] assessed the efficacy of vasopressors for the treatment of any circulatory failure in RCTs. The authors did not pool results for studies assessing septic patients alone. No mortality benefit was demonstrated for all direct comparisons between different vasopressors or vasopressor combinations. Dopamine was associated with more arrhythmias. Another review [46], focused only on the comparison of norepinephrine and dopamine in septic shock, but included observational studies as well as randomized controlled trials. It showed an advantage of norepinephrine over dopamine with regard to 28 days, all-cause mortality, RR for increased mortality with dopamine 1.12 (95% CI 1.01–1.20, I^2^ = 0%, n = 6). Vasu et al. [47] assessed norepinephrine and dopamine in septic shock, however the data quoted from the largest trial were inaccurate [15], thus introducing further bias.

Several limitations of this review should be noted. Included trials span a long period, starting before 1989 and the last published in 2012. During this period advances in support and early goal directed treatment had been introduced to improve the quality of care in ICU. However, mortality rates were unchanged from early to recent trials (weighted mean crude mortality rate 45.0%). We could not perform a meta-analysis of differences from baseline for the hemodynamic measures, which might be more easily interpreted than end values, because results were not reported as such. An individual patient data meta-analysis might provide a better understanding of the evidence accrued to date. To note, most of the trials were methodologically good or very good, however the treatment algorithm were different in regard to dosages, time to events and monitoring. The trials were diverse in reporting and statistical methods.

Future trials should use a uniformly acceptable protocol consisting of adequate methodological design, a common sepsis management algorithm (including the use of fluid resuscitation, stress dose steroids and initial hemodynamic targets). The reported outcomes should include all-cause mortality and, for patients discharged alive, length of hospital or ICU stay, length of ventilation, length of vasopressor support and AEs.

## Conclusion

These data supply further support for the use of norepinephrine over dopamine for the treatment of patients with septic shock in ICUs, given consistent reduced all-cause mortality at 28 days with supporting hemodynamic data and lower rate of major AEs and cardiac arrhythmias. This recommendation is based on data from all the RCTs published up to date, with no heterogeneity. Trials to guide recommendations for the use other vasopressors, especially non-adrenergic, are needed.

## Supporting Information

S1 Fig Funnel plot of norepinephrine vs dopamine primary outcome, Begg and Mazumdar rank correlation, Egger’s regression intercept. (DOCX)

S2 Fig Forest plots (a-r). **a,** Norepinephrine vs epinephrine, Mortality primary. **b,** Norepinephrine vs vasopressin OR terlipressin, Mortality primary. **c,** Norepinephrine vs phenylephrine, Mortality primary. **d,** Norepinephrine vs other, Mortality primary. **e,** Vasopressin / terlipressin vs other, Mortality Primary. **f,** Norepinephrine vs other, ICU (or hospital) stay. **g,** Norepinephrine vs other, 1st measurement point CVP. **h,** Norepinephrine vs vasopressin or terlipressin, 1st measurement point lactate. **i,** Norepinephrine vs dopamine, 1st measurement point urine output. **j,** Norepinephrine vs dopamine, 1st measurement point CIX. **k,** Norepinephrine vs epinephrine, 1st measurement point CIX. **l,** Norepinephrine vs other, 1st measurement point CIX. **m,** Norepinephrine vs dopamine, 1st measurement point heart rate. **n,** Norepinephrine vs other, 1st measurement point heart rate. **o,** Norepinephrine vs dopamine, 1st measurement point SVRI. **p,** Norepinephrine vs vasopressin or terlipressin, 1st measurement point SVRI. **q,** Norepinephrine vs other, 1st measurement point VIo2. **r,** Norepinephrine vs Epinephrine, 1st measurement point splanchnic CO2 difference.
(DOCX)

S1 Table Hemodynamic data reported and baseline, first and second measurement points. (DOCX)

S2 Table List of excluded studies and reason for exclusion. (DOCX)

S3 Table PRISMA 2009 checklist. (DOCX)
