# Supplementary material for: Vasopressors for the Treatment of Septic Shock: Systematic Review and Meta-Analysis
Source: PLoS One. 2015 Aug 3;10(8):e0129305. doi: 10.1371/journal.pone.0129305 (PMC4523170; doi:10.1371/journal.pone.0129305)
Supplement: S1 Fig — (DOCX) [file pone.0129305.s001.docx]

Figure 1 – Funnel plot of norepinephrine vs dopamine primary outcome


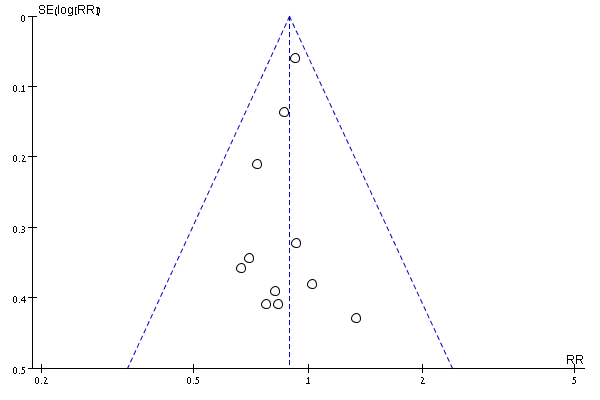


Begg and Mazumdar rank correlation

Kendall’s S statistics (P-Q) 5.000

Kendall’s tau without continuity correction

Tau 0.09091

z-value for tau 0.38925

P-value (1 tailed) 0.34855

P-value (2 tailed) 0.69709

Kendall’s tau with continuity correction

Tau 0.07273

z-value for tau 0.31140

P-value (1 tailed) 0.37775

P-value (2 tailed) 0.75550

Egger’s regression intercept

Intercept –0.30556

Standard error 0.26668

95% lower limit (2 tailed) –0.90884

95% upper limit (2 tailed) 0.29772

t-value 1.14578

df 9.0000

P -value (1 tailed) 0.14072

P -value (2 tailed) 0.28144
