# Supplementary material for: Vasopressors for the Treatment of Septic Shock: Systematic Review and Meta-Analysis
Source: PLoS One. 2015 Aug 3;10(8):e0129305. doi: 10.1371/journal.pone.0129305 (PMC4523170; doi:10.1371/journal.pone.0129305)

Figure 2 – forest plots (a-r)

1. Norepinephrine vs epinephrine, Mortality primary.


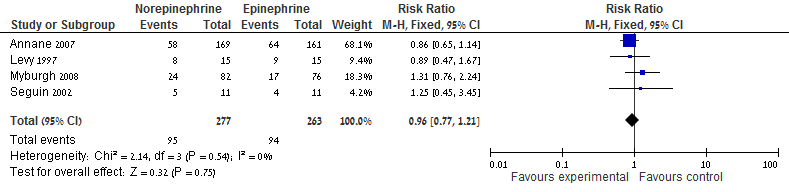


1. Norepinephrine vs vasopressin OR terlipressin, Mortality primary.


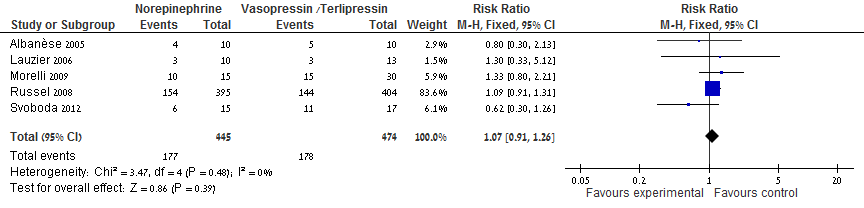


1. Norepinephrine vs phenylephrine, Mortality primary.


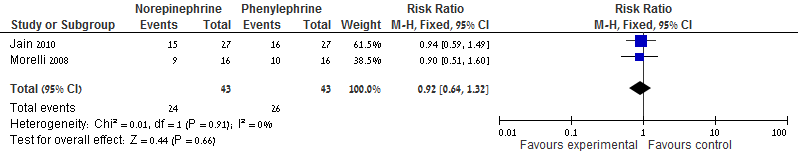


1. Norepinephrine vs other, Mortality primary.


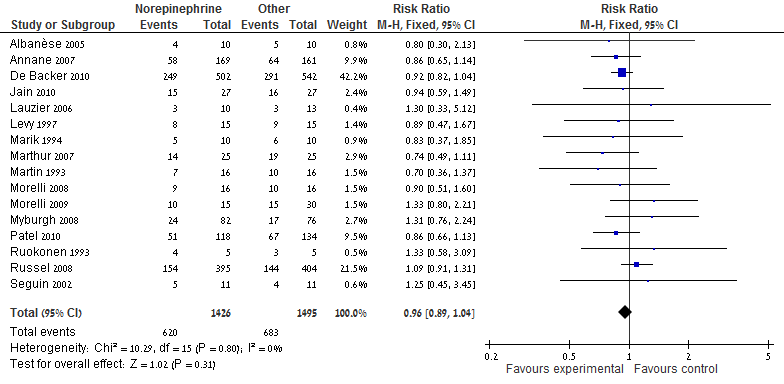


1. Vasopressin / terlipressin vs other, Mortality Primary.


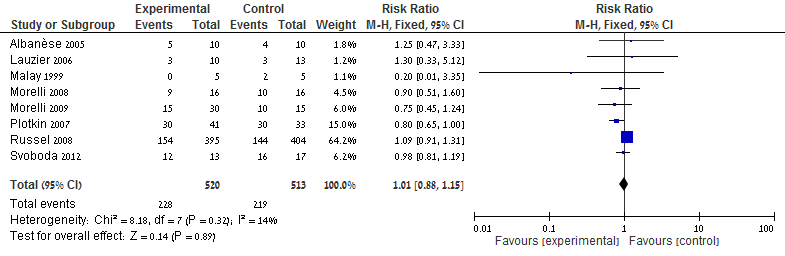


1. Norepinephrine vs other, ICU (or hospital) stay.


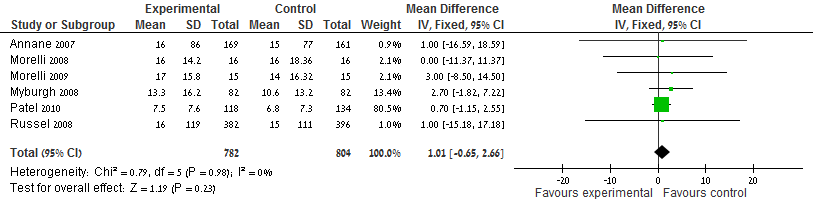


1. Norepinephrine vs other, 1st measurement point CVP.


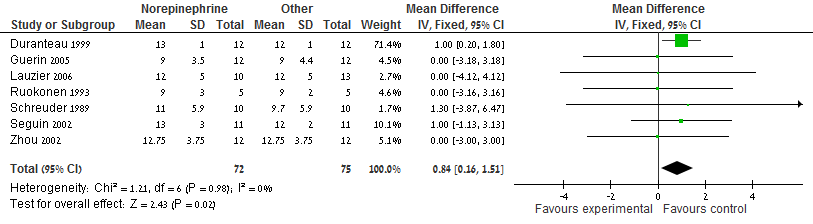


1. Norepinephrine vs vasopressin or terlipressin, 1st measurement point lactate.


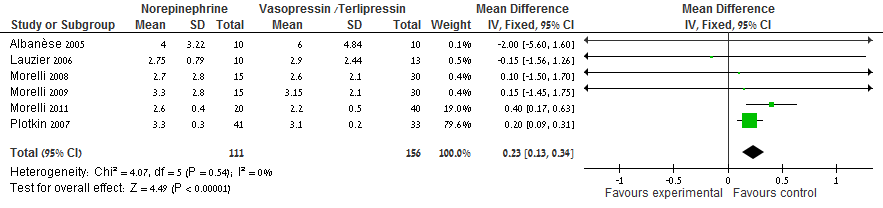


1. Norepinephrine vs dopamine, 1st measurement point urine output.


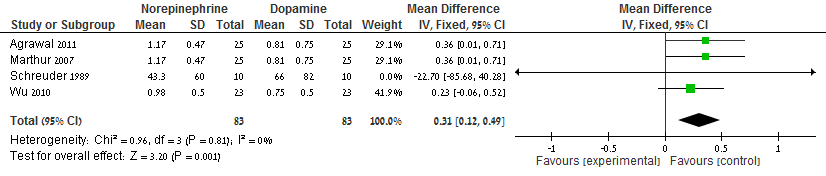


1. Norepinephrine vs dopamine, 1st measurement point CIX.


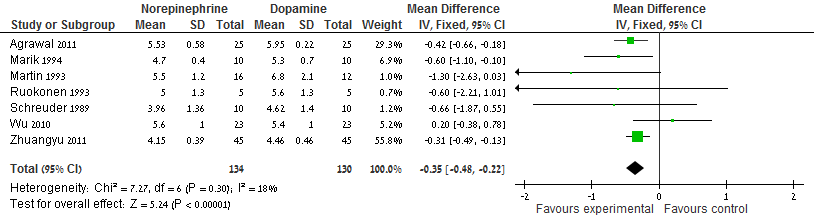


1. Norepinephrine vs epinephrine, 1st measurement point CIX.


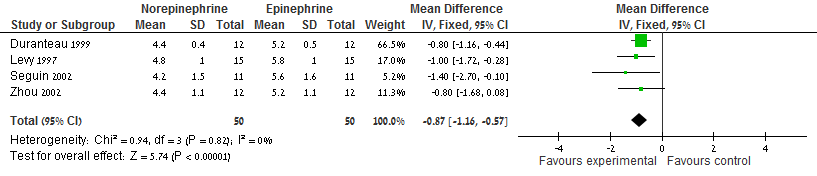


1. Norepinephrine vs other, 1st measurement point CIX.


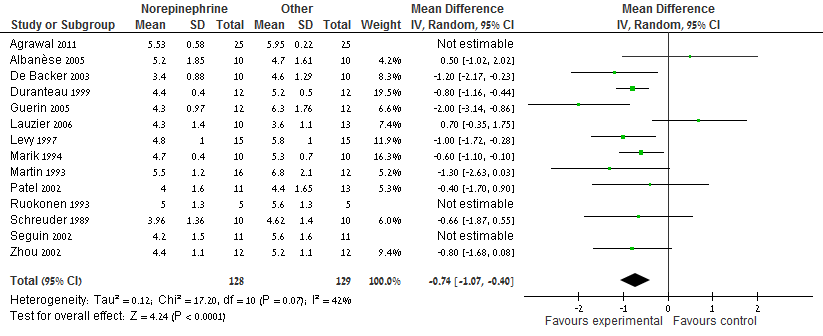


1. Norepinephrine vs dopamine, 1st measurement point heart rate.


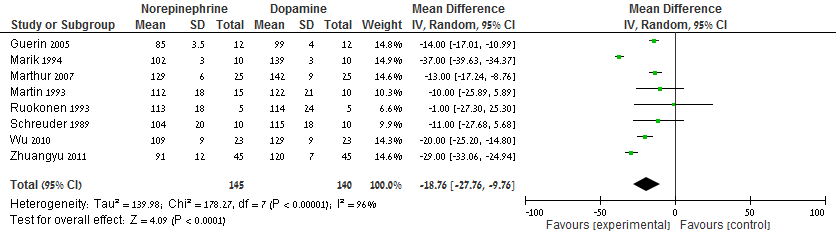


1. Norepinephrine vs other, 1st measurement point heart rate.


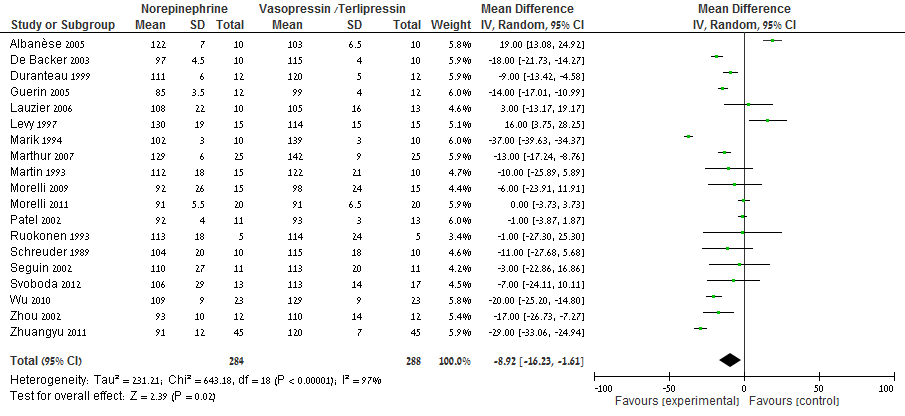


1. Norepinephrine vs dopamine, 1st measurement point SVRI.


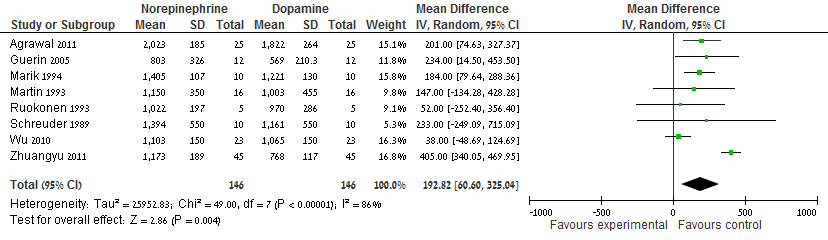


1. Norepinephrine vs vasopressin or terlipressin, 1st measurement point SVRI.


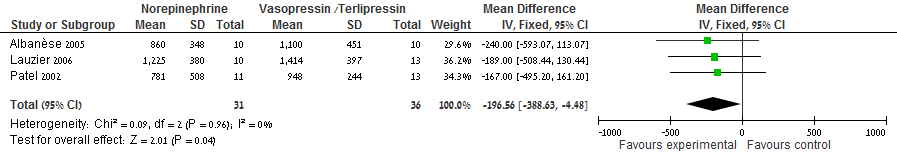


1. Norepinephrine vs other, 1st measurement point VIo_2_.


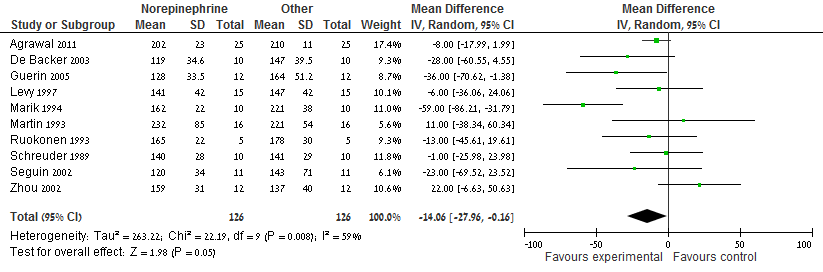


1. Norepinephrine vs Epinephrine, 1st measurement point splanchnic CO_2_ difference.


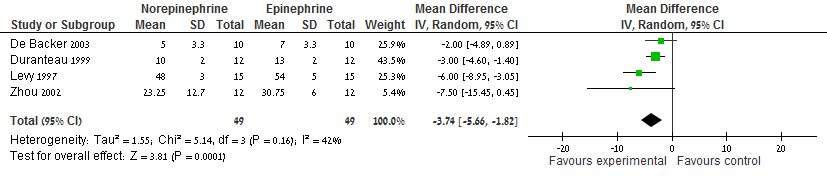

Supplement: S2 Fig — a, Norepinephrine vs epinephrine, Mortality primary. b, Norepinephrine vs vasopressin OR terlipressin, Mortality primary. c, Norepinephrine vs phenylephrine, Mortality primary. d, Norepinephrine vs other, Mortality primary. e, Vasopressin / terlipressin vs other, Mortality Primary. f, Norepinephrine vs other, ICU (or hospital) stay. g, Norepinephrine vs other, 1st measurement point CVP. h, Norepinephrine vs vasopressin or terlipressin, 1st measurement point lactate. i, Norepinephrine vs dopamine, 1st measurement point urine output. j, Norepinephrine vs dopamine, 1st measurement point CIX. k, Norepinephrine vs epinephrine, 1st measurement point CIX. l, Norepinephrine vs other, 1st measurement point CIX. m, Norepinephrine vs dopamine, 1st measurement point heart rate. n, Norepinephrine vs other, 1st measurement point heart rate. o, Norepinephrine vs dopamine, 1st measurement point SVRI. p, Norepinephrine vs vasopressin or terlipressin, 1st measurement point SVRI. q, Norepinephrine vs other, 1st measurement point VIo2. r, Norepinephrine vs Epinephrine, 1st measurement point splanchnic CO2 difference. (DOCX) [file pone.0129305.s002.docx]
