# Supplementary material for: Vasopressors for the Treatment of Septic Shock: Systematic Review and Meta-Analysis
Source: PLoS One. 2015 Aug 3;10(8):e0129305. doi: 10.1371/journal.pone.0129305 (PMC4523170; doi:10.1371/journal.pone.0129305)
Supplement: S1 Table — (DOCX) [file pone.0129305.s003.docx]

**Supplemental material**

Table 1: hemodynamic data reported and baseline, first and second measurement points

| Author, year  intervention arms | MAP at randomization (mmHg) | First measurement point MAP (mmHg) | Second measurement point MAP (mmHg) | Lactate at randomization (mmol/l) | First measurement point Lactate (mmol/l) | Second measurement point Lactate (mmol/l) | CVP at randomization (mmHg) | First measurement point CVP (mmHg) | Second measurement point CVP (mmHg) |
| --- | --- | --- | --- | --- | --- | --- | --- | --- | --- |
| Agrawal 2011 |  |  |  |  |  |  |  |  |  |
| Norepinephrine | 5.45 ± 0.57 | 5.53 ± 0.58 | NS | NS | NS | NS | NS | NS | NS |
| Dopamine | 5.28 ± 0.53 | 5.95 ± 0.22 | NS | NS | NS | NS | NS | NS | NS |
| Albanèse 2005 |  |  |  |  |  |  |  |  |  |
| Terlipressin | 54 (48–62) | 72  (62–82) | 69  (63–83) | 4.5 | NS | 6 ± 4.84 | NS | NS | NS |
| Norepinephrine | 54 (49–61) | 72  (62–81) | 72  (61–82) | 4.4 | NS | 4 ± 3.22 | NS | NS | NS |
| Annane 2007 |  |  |  |  |  |  |  |  |  |
| Norepinephrine + dobutamine | 68 (19) | NS | NS | 3.3 (2.1–5.1) | NS | NS | NS | NS | NS |
| epinephrine | 70 (19) | NS | NS | 2.9 (1.7–5.0) | NS | NS | NS | NS | NS |
| Chen 2012 |  |  |  |  |  |  |  |  |  |
| Norepinephrine | NS | NS | NS | NS | NS | NS | NS | NS | NS |
| Dopamine | NS | NS | NS | NS | NS | NS | NS | NS | NS |
| De Backer 2003 |  |  |  |  |  |  |  |  |  |
| Epinephrine | NS | 74  (72–76) | NS | NS | 4.3  (2.0–5.3) | NS | NS | NS | NS |
| Norepinephrine | NS | 73  (72–75) | NS | NS | 3.2  (1.3–3.8) | NS | NS | NS | NS |
| De Backer 2010 |  |  |  |  |  |  |  |  |  |
| Norepinephrine | 58 ± 1 | NS | NS | NS | NS | NS | 12.8 ± 0.5 | NS | NS |
| Dopamine | 58 ± 1 | NS | NS | NS | NS | NS | 12.5 ± 0.7 | NS | NS |
| Duranteau 1999 |  |  |  |  |  |  |  |  |  |
| Epinephrine | NS | 74 ± 1 | NS | NS | NS | NS | NS | 12 ± 1 | NS |
| Norepinephrine | NS | 74 ± 1 | NS | NS | NS | NS | NS | 13 ± 1 | NS |
| Norepinephrine+ dobutamine | NS | 74 ± 1 | NS | NS | NS | NS | NS | 12 ± 1 | NS |
| Guérin 2005 |  |  |  |  |  |  |  |  |  |
| Norepinephrine | NS | 86  (84–90) | NS | NS | NS | NS | NS | 9 (7–11) | NS |
| Dopamine | NS | 84  (79–92) | NS | NS | NS | NS | NS | 9 (7–12) | NS |
| High 2008 |  |  |  |  |  |  |  |  |  |
| Norepinephrine | NS | NS | NS | NS | NS | NS | NS | NS | NS |
| Dopamine | NS | NS | NS | NS | NS | NS | NS | NS | NS |
| Jain 2010 |  |  |  |  |  |  |  |  |  |
| Norepinephrine | 47.55 ± 4.3 | NS | 76.1 ± 7.4 | 3.4 ± 0.74 | NS | 2.9 ± 0.35 | NS | NS | NS |
| Phenylephrine | 48.96 ± 3.36 | NS | 77.8 ± 6.6 | 3.44 ± 0.64 | NS | 2.87 ± 0.39 | NS | NS | NS |
| Lauzier 2006 |  |  |  |  |  |  |  |  |  |
| Norepinephrine | 68 ± 10 | 72 ± 5 | 72 ± 8 # | 3.33 ± 1.75 | NS | 2.75 ± 0.79 # | 13 ± 7 | 12±5 | 12±6 # |
| Vasopressin | 72 ± 7 | 74 ± 8 | 75 ± 7 # | 2.87 ± 0.75 | NS | 2.90 ± 2.44 # | 10 ± 4 | 12±5 | 12±6 # |
| Levy 1997 |  |  |  |  |  |  |  |  |  |
| Dobutamine-norepinephrine | 60 ± 8 | 87 ± 8 | 86 ± 8 | 3.1 ± 1.5 | 3.1 ± 1.0 | 5.9 ± 1.0 | NS | NS | NS |
| epinephrine | 60 ± 8 | 89 ± 8 | 93 ± 8 | 3.1 ± 1.5 | 4.8 ± 1.5 | 2.7 ± 1.0 | NS | NS | NS |
| Liu 2010 |  |  |  |  |  |  |  |  |  |
| Norepinephrine | NS | NS | NS | 4.96 ± 1.34 | 2.97 ± 1.1 | 2.16 ± 0.7 | NS | NS | NS |
| Dopamine | NS | NS | NS | 4.68 ± 1.37 | 3.25 ± 1.0 | 2.88 ± 0.9 | NS | NS | NS |
| Malay 1999 |  |  |  |  |  |  |  |  |  |
| Vasopressin | 64 ± 6 | 80 ± 8 | 81 ±5 | NS | NS | NS | NS | NS | NS |
| Placebo | 66 ± 6 | 68 ±5 | 80 ± 5 | NS | NS | NS | NS | NS | NS |
| Marik 1994 |  |  |  |  |  |  |  |  |  |
| Norepinephrine | 65 ± 4 | 87 ± 4 | NS | 1.8 ± 0.5 | 1.9 ± 0.43 | NS | NS | NS | NS |
| Dopamine | 63 ± 2 | 87 ± 3 | NS | 2.2 ± 0.4 | 2.1 ± 0.4 | NS | NS | NS | NS |
| Marthur 2007 |  |  |  |  |  |  |  |  |  |
| Norepinephrine | NS | NS | NS | NS | NS | NS | NS | NS | NS |
| Dopamine | NS | NS | NS | NS | NS | NS | NS | NS | NS |
| Martin 1993 |  |  |  |  |  |  |  |  |  |
| Norepinephrine | 54 ± 10 | 89 ± 13 | 91 | 4.8 ± 1.6 | 4.4 ± 1.8 | 2.9 ± 0.8 | NS | NS | NS |
| Dopamine | 53 ± 8 | 94 ± 12 | 96 ± 5 | 4.8 ± 3.2 | 4.2 ± 2.0 | 3.8 ± 2.0 | NS | NS | NS |
| Morelli 2008 |  |  |  |  |  |  |  |  |  |
| Phenylephrine | 54 | NS | 68 # | 2.8 | NS | 2.7 ± 2.6 # | 13 ± 3 | NS | 15 ± 3 # |
| Norepinephrine | 56 | NS | 72 # | 2.8 | NS | 2.7 ± 2.6 # | 13 ± 3 | NS | 14 ± 3 # |
| Morelli 2009 |  |  |  |  |  |  |  |  |  |
| Terlipressin | 53 ± 6 | NS | 70 ± 3 # | 3.1 ± 1.8 | NS | 2.9 ± 1.9 # | 11 ± 3 | NS | 12 ± 3 # |
| Vasopressin | 53 ± 4 | NS | 70 ± 3 # | 3.0 ± 2.4 | NS | 3.2 ± 2.3 # | 12 ± 3 | NS | 15 ± 3 # |
| Norepinephrine | 54 ± 3 | NS | 70 ± 4 # | 3.1 ± 2.2 | NS | 3.3 ± 2.8 # | 12 ± 3 | NS | 13 ± 3 # |
| Morelli 2011 |  |  |  |  |  |  |  |  |  |
| Terlipressin | 71 (68 - 75) | NS | 74  (72 - 75) | 1.8 (1.2 - 2.9) | NS | 2.1  (1.5 - 2.8) | 13 (10 - 15) | NS | 13  (10 - 15) |
| Vasopressin | 72 (69 - 75) | NS | 72  (68 - 75) | 2.3 (1.4 - 3.6) | NS | 2.3  (1.4 - 3.6) | 15 (12 - 17) | NS | 14  (11 - 16) |
| Norepinephrine | 71 (68 - 75) | NS | 74  (68 - 75) | 2.5 (1.9 - 3.0) | NS | 2.6  (2.1 - 3.7) | 13 (10 - 14) | NS | 11  (10 - 14) |
| Myburgh 2008 |  |  |  |  |  |  |  |  |  |
| Norepinephrine | 66.3 ± 9.8 | NS | NS | 2.4 ± 2.7 | NS | NS | NS | NS | NS |
| epinephrine | 65.9 ± 11.4 | NS | NS | 2.7 ± 2.5 | NS | NS | NS | NS | NS |
| Patel 2002 |  |  |  |  |  |  |  |  |  |
| Norepinephrine | 68 (65 - 70) | 67  (61 - 70) | NS | NS | NS | NS | NS | NS | NS |
| Vasopressin | 69 (65 - 72) | 69  (65 - 70) | NS | NS | NS | NS | NS | NS | NS |
| Patel 2010 |  |  |  |  |  |  |  |  |  |
| Norepinephrine | NS | NS | NS | NS | NS | NS | NS | NS | NS |
| Dopamine | NS | NS | NS | NS | NS | NS | NS | NS | NS |
| Plotkin 2007 |  |  |  |  |  |  |  |  |  |
| Terlipressin | NS | NS | NS | 3.6 ± 0.4 | 3.5 ± 0.2 | 3.3 ± 0.3 | NS | NS | NS |
| Dopamine | NS | NS | NS | 3.2 ±0.3 | 2.6 ± 0.4 | 3.1 ± 0.2 | NS | NS | NS |
| Ruokonen 1993 |  |  |  |  |  |  |  |  |  |
| Norepinephrine | 55 ± 1 | 70 ± 4 | NS | 2.1 ± 1.1 | 1.9 ± 0.9 | NS | 8 ± 2 | 8 ± 3 | NS |
| Dopamine | 59 ± 2 | 73 ± 6 | NS | 1.3 ± 0.64 | 1.2 ± 0.6 | NS | 10 ± 3 | 9 ± 4 | NS |
| Russel 2008 |  |  |  |  |  |  |  |  |  |
| Norepinephrine | 73 ± 10 | NS | NS | 3.5 ± 3.0 | NS | NS | NS | NS | NS |
| Vasopressin | 72 ± 9 | NS | NS | 3.5 ± 3.2 | NS | NS | NS | NS | NS |
| Schreuder 1989 |  |  |  |  |  |  |  |  |  |
| Norepinephrine | 56.8 ± 9.7 | 26.9 ± 2.6 | NS | 2.3 ± 1.9 | 2.3 ± 1.9 | NS | 7.2 4.3 | 11.1 ± 5.9 | NS |
| Dopamine | 56.8 ± 9.7 | 25.5 ± 4.6 | NS | 2.3 ± 1.9 | 2.6 ± 2.2 | NS | 7.2 4.3 | 9.7 ± 5.9 | NS |
| Seguin 2002 |  |  |  |  |  |  |  |  |  |
| Dobutamine-norepinephrine | 51 ± 8 | 77 ± 5 | NS | 5.3 ± 4.3 | 5.0 ± 3.5 | NS | 13 ± 2 | 13 ± 3 | NS |
| epinephrine | 54 ± 8 | 78 ± 3 | NS | 3.9 ± 2.6 | 4.8 ± 2.9 | NS | 13 ± 3 | 12 ± 2 | NS |
| Svoboda 2012 |  |  |  |  |  |  |  |  |  |
| Norepinephrine | 71.4 ± 9.9 | NS | NS | NS | NS | NS | NS | NS | NS |
| Terlipressin | 74.4 ± 9.8 | NS | NS | NS | NS | NS | NS | NS | NS |
| Wu 2010 |  |  |  |  |  |  |  |  |  |
| Norepinephrine | 52.2 ± 12.4 | 78.2 | NS | 5.9 ± 1.8 | NS | NS | NS | NS | NS |
| Dopamine | 50.9 ± 11.9 | 50.9 | NS | 6.1 ± 1.5 | NS | NS | NS | NS | NS |
| Zhou 2002 |  |  |  |  |  |  |  |  |  |
| Epinephrine | 60 | 87 ± 6 | NS | 2.6 ± 2.2 | 2.3 ±1.6 | NS | NS | 12.75 ± 3.75 | NS |
| Norepinephrine | 60 | 93 ± 8 | NS | 2.6 ± 2.2 | 1.6 ± 1.2 | NS | NS | 12.75 ± 3.75 | NS |
| Norepinephrine+Dubotamine | 60 | 90 | NS | 2.6 ± 2.2 | 1.1 ± 0.7 | NS | NS | 12.75 ± 3.5 | NS |
| Zhuangyu 2011 |  |  |  |  |  |  |  |  |  |
| Norepinephrine | NS | NS | NS | NS | NS | NS | NS | NS | NS |
| Dopamine | NS | NS | NS | NS | NS | NS | NS | NS | NS |

Table 1 continued: hemodynamic data reported and baseline, first and second measurement points, continues

| Author, year  intervention arms | First measurement point urine output (cc/mg/hr) | Second measurement point urine output (cc/mg/hr) | First measurement point ScvO_2_ (%) | Second measurement point ScvO_2_ (%) | First measurement point heart rate | Second measurement point heart rate | First measurement point VIo_2_ | Second measurement point VIo_2_ | ICU stay |
| --- | --- | --- | --- | --- | --- | --- | --- | --- | --- |
| Agrawal 2011 |  |  |  |  |  |  |  |  |  |
| Norepinephrine | 1.17 ± 0.47 | NS | NS | NS | NS | NS | NS | 202 ± 23 | NS |
| Dopamine | 0.81 ± 0.75 | NS | NS | NS | NS | NS | NS | 210 ± 11 | NS |
| Albanèse 2005 |  |  |  |  |  |  |  |  |  |
| Terlipressin | NS | 1 | NS | NS | 122 (81–133) | NS | NS | NS | NS |
| Norepinephrine | NS | 1.1 | NS | NS | 103 (74–118) | NS | NS | NS | NS |
| Annane 2007 |  |  |  |  |  |  |  |  |  |
| Norepinephrine + dobutamine | NS | NS | NS | NS | NS | NS | NS | NS | 16 (6–32) |
| epinephrine | NS | NS | NS | NS | NS | NS | NS | NS | 15 (7–31) |
| Chen 2012 |  |  |  |  |  |  |  |  |  |
| Norepinephrine | NS | NS | NS | NS | NS | NS | NS | NS | NS |
| Dopamine | NS | NS | NS | NS | NS | NS | NS | NS | NS |
| De Backer 2003 |  |  |  |  |  |  |  |  |  |
| Epinephrine | NS | NS | 72.3 (69–73.9) | NS | 115 (105–121) | NS | 147 ± 39.5 | NS | NS |
| Norepinephrine | NS | NS | 72.5 (71–74.5) | NS | 97 (84–105) | NS | 119 ± 34.6 | NS | NS |
| De Backer 2010 |  |  |  |  |  |  |  |  |  |
| Norepinephrine | *1200 ± 100 | NS | 62.5 ± 2 | NS | NS | NS | NS | NS | NS |
| Dopamine | *1350 ± 100 | NS | 62.4 ± 2 | NS | NS | NS | NS | NS | NS |
| Duranteau 1999 |  |  |  |  |  |  |  |  |  |
| Epinephrine | NS | NS | NS | NS | 120 ± 5 | NS | NS | NS | NS |
| Norepinephrine | NS | NS | NS | NS | 111 ± 6 | NS | NS | NS | NS |
| Norepinephrine+Dubotamine | NS | NS | NS | NS | 120 ± 4 | NS | NS | NS | NS |
| Guérin 2005 |  |  |  |  |  |  |  |  |  |
| Norepinephrine | NS | NS | 64.1 (55–68.6) | NS | 85 (78–90) | NS | 128 ± 33.5 | NS | NS |
| Dopamine | NS | NS | 69.7 (65.9–72) | NS | 99 (93–109) | NS | 164 ± 51.2 | NS | NS |
| High 2008 |  |  |  |  |  |  |  |  |  |
| Norepinephrine | NS | NS | NS | NS | NS | NS | NS | NS | NS |
| Dopamine | NS | NS | NS | NS | NS | NS | NS | NS | NS |
| Jain 2010 |  |  |  |  |  |  |  |  |  |
| Norepinephrine | NS | NS | NS | 66.88 ± 1.76 | NS | NS | NS | 200 ± 8.0 | NS |
| Phenylephrine | NS | NS | NS | 67.4 ± 2.04 | NS | NS | NS | 195 ± 6.8 | NS |
| Lauzier 2006 |  |  |  |  |  |  |  |  |  |
| Norepinephrine | NS | NS | NS | NS | 108 ± 22 | 104 ± 22 # | NS | 133 ± 26 # | NS |
| Vasopressin | NS | NS | NS | NS | 105 ±16 | 100 ± 15 # | NS | 133 ± 26 # | NS |
| Levy 1997 |  |  |  |  |  |  |  |  |  |
| Dobutamine-norepinephrine | NS | NS | NS | NS | 130 ± 19 | 129 ± 15 | 141 ± 42 | 159 ± 46 | NS |
| epinephrine | NS | NS | NS | NS | 114 ± 15 | 109 ± 11 | 147 ± 42 | 156 ± 50 | NS |
| Liu 2010 |  |  |  |  |  |  |  |  |  |
| Norepinephrine | NS | NS | NS | NS | NS | NS | NS | NS | NS |
| Dopamine | NS | NS | NS | NS | NS | NS | NS | NS | NS |
| Malay 1999 |  |  |  |  |  |  |  |  |  |
| Vasopressin | NS | NS | NS | NS | 106 ± 8 | NS | NS | NS | NS |
| Placebo | NS | NS | NS | NS | 128 ± 14 | NS | NS | NS | NS |
| Marik 1994 |  |  |  |  |  |  |  |  |  |
| Norepinephrine | NS | NS | NS | NS | 102 ± 3 | NS | 162 ± 22 | NS | NS |
| Dopamine | NS | NS | NS | NS | 139 ± 3 | NS | 221 ± 38 | NS | NS |
| Marthur 2007 |  |  |  |  |  |  |  |  |  |
| Norepinephrine | 0.37 ± 0.11 | 1.17 ± 0.47 | NS | NS | 129 ± 6 | NS | NS | 202 ± 23 | NS |
| Dopamine | 0.34 ± 0.28 | 1.17 ± 0.47 | NS | NS | 142 ± 9 | NS | NS | 210 ± 11 | NS |
| Martin 1993 |  |  |  |  |  |  |  |  |  |
| Norepinephrine | NS | NS | NS | NS | 112 ± 18 | 123 (79–137) | 232 ± 85 | 235 ± 74 | NS |
| Dopamine | NS | NS | NS | NS | 122 ± 21 | 99 (68–103) | 221 ± 54 | 193 ± 51 | NS |
| Morelli 2008 |  |  |  |  |  |  |  |  |  |
| Phenylephrine | NS | NS | NS | 67 ± 9 # | NS | NS | NS | 161 ± 58 # | 16 (10 - 24) |
| Norepinephrine | NS | NS | NS | 67 ± 10 # | NS | NS | NS | 150 ± 41 # | 16 (7 - 25) |
| Morelli 2009 |  |  |  |  |  |  |  |  |  |
| Terlipressin | NS | NS | NS | 59 ± 11 # | 85 ± 19 | 83 ± 21 | NS | 184 ± 49 # | 14 (9 - 25) |
| Vasopressin | NS | NS | NS | 65 ± 11 # | 98 ± 24 | 97 ± 27 | NS | 173 ± 59 # | 17 (5 - 27) |
| Norepinephrine | NS | NS | NS | 66 ± 10 # | 92 ± 26 | 99 ± 29 | NS | 147 ± 35 # | 17(7 - 23) |
| Morelli 2011 |  |  |  |  |  |  |  |  |  |
| Terlipressin | NS | NS | NS | 70 (66 - 77) # | NS | NS | NS | NS | NS |
| Vasopressin | NS | NS | NS | 70 (59 - 77) # | NS | NS | NS | NS | NS |
| Norepinephrine | NS | NS | NS | 67 (59 - 72) # | NS | NS | NS | NS | NS |
| Myburgh 2008 |  |  |  |  |  |  |  |  |  |
| Norepinephrine | *217.9 ± 213.3 | NS | NS | NS | NS | NS | NS | NS | 13.3 ± 16.2 |
| epinephrine | *176.8 ± 218.4 | NS | NS | NS | NS | NS | NS | NS | 10.6 ± 13.2 |
| Patel 2002 |  |  |  |  |  |  |  |  |  |
| Norepinephrine | 18 | NS | NS | NS | 92 (83 -100) | NS | NS | NS | NS |
| Vasopressin | 65 | NS | NS | NS | 93 (91 -100) | NS | NS | NS | NS |
| Patel 2010 |  |  |  |  |  |  |  |  |  |
| Norepinephrine | NS | NS | NS | NS | NS | NS | NS | NS | 7.5 ± 7.6 |
| Dopamine | NS | NS | NS | NS | NS | NS | NS | NS | 6.8 ± 7.3 |
| Plotkin 2007 |  |  |  |  |  |  |  |  |  |
| Terlipressin | NS | NS | NS | NS | NS | NS | NS | NS | NS |
| Dopamine | NS | NS | NS | NS | NS | NS | NS | NS | NS |
| Ruokonen 1993 |  |  |  |  |  |  |  |  |  |
| Norepinephrine | NS | NS | NS | NS | 113 ± 18 | NS | 165 ± 22 | NS | NS |
| Dopamine | NS | NS | NS | NS | 114 ± 24 | NS | 178 ± 30 | NS | NS |
| Russel 2008 |  |  |  |  |  |  |  |  |  |
| Norepinephrine | NS | NS | NS | NS | NS | NS | NS | NS | 16 (8–32) |
| Vasopressin | NS | NS | NS | NS | NS | NS | NS | NS | 15 (7–29) |
| Schreuder 1989 |  |  |  |  |  |  |  |  |  |
| Norepinephrine | *43 ± 60 | NS | NS | NS | 104 ± 20 | NS | 140 ± 28 | NS | NS |
| Dopamine | *66 ± 82 | NS | NS | NS | 115 ± 18 | NS | 141 ± 29 | NS | NS |
| Seguin 2002 |  |  |  |  |  |  |  |  |  |
| Dobutamine-norepinephrine | NS | NS | 71 ± 10 | NS | 110 ± 27 | NS | 120 ± 34 | NS | NS |
| epinephrine | NS | NS | 74 ± 11 | NS | 113 ± 20 | NS | 143 ± 71 | NS | NS |
| Svoboda 2012 |  |  |  |  |  |  |  |  |  |
| Norepinephrine | NS | NS | NS | NS | 106 ± 29 | NS | NS | NS | NS |
| Terlipressin | NS | NS | NS | NS | 113 ± 14 | NS | NS | NS | NS |
| Wu 2010 |  |  |  |  |  |  |  |  |  |
| Norepinephrine | 0.98 ± 0.32 | NS | NS | NS | 109 | NS | NS | NS | NS |
| Dopamine | 0.75 ± 0.28 | NS | NS | NS | 129 | NS | NS | NS | NS |
| Zhou 2002 |  |  |  |  |  |  |  |  |  |
| Epinephrine | NS | NS | NS | NS | 110 ± 14 | NS | 137 ± 40 | NS | NS |
| Norepinephrine | NS | NS | NS | NS | 93 ± 10 | NS | 159 ± 31 | NS | NS |
| Norepinephrine+Dubotamine | NS | NS | NS | NS | 87 ± 9 | NS | 134 ± 35 | NS | NS |
| Zhuangyu 2011 |  |  |  |  |  |  |  |  |  |
| Norepinephrine | NS | NS | NS | NS | 91 ± 12 | NS | NS | NS | NS |
| Dopamine | NS | NS | NS | NS | 120 ± 7 | NS | NS | NS | NS |

NS – not stated; CVP – central venous pressure; MAP – mean arterial pressure; ScvO_2_ – central venous saturation;

Results presented as either mean, mean ± standard deviation or median (interquartile range).

* There was insufficient data to calculate urine output per kg/hr from the publication

# taken at 12 hours
