# Supplementary material for: Vasopressors for the Treatment of Septic Shock: Systematic Review and Meta-Analysis
Source: PLoS One. 2015 Aug 3;10(8):e0129305. doi: 10.1371/journal.pone.0129305 (PMC4523170; doi:10.1371/journal.pone.0129305)
Supplement: S2 Table — (DOCX) [file pone.0129305.s004.docx]

Table 2 – list of excluded studies and reason for exclusion

Reason for exclusion

Non-randomized [1-11]

No sepsis [12-14]

Double publications [15-19]

Insufficient data [20-28]

Incompatible vasopressors comparison:

1. Same vasopressor [29]
2. Dopexamine [30-31]
3. Dobutamine alone [32]
4. Amrinone, enoximone, angiotensin [33-36]

References for excluded studies

1. Day, N.P., et al., *Effects of dopamine and epinephrine infusions on renal hemodynamics in severe malaria and severe sepsis.* Crit Care Med, 2000. **28**(5): p. 1353-62.

2. Dupeyron, J.P., et al., *[Use of dobutamine in the treatment of septic shock].* Anesth Analg (Paris), 1977. **34**(5): p. 917-27.

3. Hannemann, L., et al., *Comparison of dopamine to dobutamine and norepinephrine for oxygen delivery and uptake in septic shock.* Crit Care Med, 1995. **23**(12): p. 1962-70.

4. Klinzing, S., et al., *High-dose vasopressin is not superior to norepinephrine in septic shock.* Crit Care Med, 2003. **31**(11): p. 2646-50.

5. Landry, D.W., et al., *Vasopressin pressor hypersensitivity in vasodilatory septic shock.* Crit Care Med, 1997. **25**(8): p. 1279-82.

6. Lauro, E. and L. Zeni, *[Clinical trial of dopamine in the treatment of shock].* Minerva Anestesiol, 1977. **43**(5): p. 307-22.

7. Martinez, J.T., G. Fernandez, and H. Vazquez-Leon, *Clinical evaluation of new therapeutic concepts in septic shock.* Obstet Gynecol, 1966. **27**(2): p. 296-301.

8. Meier-Hellmann, A., et al., *Epinephrine impairs splanchnic perfusion in septic shock.* Crit Care Med, 1997. **25**(3): p. 399-404.

9. Morelli, A., et al., *Effects of short-term simultaneous infusion of dobutamine and terlipressin in patients with septic shock: the DOBUPRESS study.* Br J Anaesth, 2008. **100**(4): p. 494-503.

10. Regnier, B., et al., *[Comparative study of the effects of dobutamine and dopamine in septic shock].* Ann Anesthesiol Fr, 1978. **19**(10): p. 859-62.

11. Wilson, R.F., W.J. Sibbald, and J.L. Jaanimagi, *Hemodynamic effects of dopamine in critically ill septic patients.* J Surg Res, 1976. **20**(3): p. 163-72.

12. Boyd, O., et al., *A comparison of the efficacy of dopexamine and dobutamine for increasing oxygen delivery in high-risk surgical patients.* Anaesth Intensive Care, 1995. **23**(4): p. 478-84.

13. Duke, G.J., J.H. Briedis, and R.A. Weaver, *Renal support in critically ill patients: low-dose dopamine or low-dose dobutamine?* Crit Care Med, 1994. **22**(12): p. 1919-25.

14. Richer, M., S. Robert, and M. Lebel, *Renal hemodynamics during norepinephrine and low-dose dopamine infusions in man.* Crit Care Med, 1996. **24**(7): p. 1150-6.

15. Gordon, A.C., et al., *The effects of vasopressin on acute kidney injury in septic shock.* Intensive Care Med, 2010. **36**(1): p. 83-91.

16. Russell, J.A., et al., *Interaction of vasopressin infusion, corticosteroid treatment, and mortality of septic shock.* Crit Care Med, 2009. **37**(3): p. 811-8.

17. Schmoelz, M., et al., *Effects of Dopexamine, Dopamine or Placebo on Thyroid Function in Patients with Septic Shock*, in *Anesthesiology* 2004.

18. Torgersen, C., et al., *Plasma copeptin levels before and during exogenous arginine vasopressin infusion in patients with advanced vasodilatory shock.* Minerva Anestesiol, 2010.

19. Dunser, M.W., et al., *Does arginine vasopressin influence the coagulation system in advanced vasodilatory shock with severe multiorgan dysfunction syndrome?* Anesth Analg, 2004. **99**(1): p. 201-6.

20. Dunser, M.W., et al., *Arginine vasopressin and serum nitrite/nitrate concentrations in advanced vasodilatory shock.* Acta Anaesthesiol Scand, 2004. **48**(7): p. 814-9.

21. Day, N.P., et al., *The effects of dopamine and adrenaline infusions on acid-base balance and systemic haemodynamics in severe infection.* Lancet, 1996. **348**(9022): p. 219-23.

22. Dunser, M.W., et al., *Arginine vasopressin in advanced vasodilatory shock: a prospective, randomized, controlled study.* Circulation, 2003. **107**(18): p. 2313-9.

23. Irlbeck, M., et al., *Dopexamine Versus Dopamine or Placebo in Patients with Septic Shock and Continuous Norepinephrine - Infusion*, in *Anesthesiology*. 2003.

24. Kinstner, C., et al., *Infusion of Arginine-Vasopressin (AVP) Enhances Blood Pressure and Renal Function While Preserving Cerebral and Splanchnic Perfusion in Patients in Septic Shock*, in *Anesthesiology*. 2002.

25. Majerus, T.C., P. Chodoff, and C.O. Borel, *Dopamine and dobutamine in septic shock. A comparison.* Arch Int Physiol Biochim, 1984. **92**(4): p. S65-7.

26. Schmoelz, M., et al., *Dopexamine and Dopamine Decrease Serum Lactate Concentrations in Norepinephrine Treated Septic Shock*, in *Anesthesiology*. 2004.

27. Worthley, L.I., P. Tyler, and J.L. Moran, *A comparison of dopamine, dobutamine and isoproterenol in the treatment of shock.* Intensive Care Med, 1985. **11**(1): p. 13-9.

28. Wu, L.J., et al., *[Effect of dopamine and norepinephrine on hemodynamics and tissue oxygenation of patients with septic shock].* Zhongguo Wei Zhong Bing Ji Jiu Yi Xue, 2008. **20**(1): p. 18-22.

29. Torgersen, C., et al., *Comparing two different arginine vasopressin doses in advanced vasodilatory shock: a randomized, controlled, open-label trial.* Intensive Care Med, 2010. **36**(1): p. 57-65.

30. Morelli, A., et al., *Effects of short-term simultaneous infusion of dobutamine and terlipressin in patients with septic shock: the DOBUPRESS study.* Br J Anaesth, 2008. **100**(4): p. 494-503.

31. Schmoelz, M., et al., *Comparison of systemic and renal effects of dopexamine and dopamine in norepinephrine-treated septic shock.* J Cardiothorac Vasc Anesth, 2006. **20**(2): p. 173-8.

32. Seguin, P., et al., *Dopexamine and norepinephrine versus epinephrine on gastric perfusion in patients with septic shock: a randomized study [NCT00134212].* Crit Care, 2006. **10**(1): p. R32.

33. Neviere, R., et al., *The contrasting effects of dobutamine and dopamine on gastric mucosal perfusion in septic patients.* Am J Respir Crit Care Med, 1996. **154**(6 Pt 1): p. 1684-8.

34. Hernandez, G., et al., *[Acute effect of dobutamine and amrinone on hemodynamics and splanchnic perfusion in septic shock patients].* Rev Med Chil, 1999. **127**(6): p. 660-6.

35. Kern, H., et al., *Enoximone in contrast to dobutamine improves hepatosplanchnic function in fluid-optimized septic shock patients.* Crit Care Med, 2001. **29**(8): p. 1519-25.

36. Singh, S. and R.P. Malhotra, *Comparative study of angiotensin and nor-adrenaline in hypotensive states (shock).* J Assoc Physicians India, 1966. **14**(11): p. 639-45.
